# Genome Sequences of Five *Oenococcus oeni* Strains Isolated from Nero Di Troia Wine from the Same Terroir in Apulia, Southern Italy

**DOI:** 10.1128/genomeA.01077-14

**Published:** 2014-10-23

**Authors:** Vittorio Capozzi, Pasquale Russo, Antonella Lamontanara, Luigi Orrù, Luigi Cattivelli, Giuseppe Spano

**Affiliations:** a Department of Agriculture, Food and Environment Sciences, University of Foggia, Foggia, Italy; b Consiglio, Per la Ricerca e Sperimentazione in Agricoltura (CRA), Genomics Research Centre, Fiorenzuola d’Arda, Italy

## Abstract

*Oenococcus oeni* is the principal lactic acid bacterium responsible for malolactic fermentation in wine. Here, we announce the genome sequences of five *O. oeni* strains isolated from Nero di Troia wine undergoing spontaneous malolactic fermentation, and we report, for the first time, several genome sequences of strains isolated from the same terroir.

## GENOME ANNOUNCEMENT

*Oenococcus oeni* is the main species of lactic acid bacteria (LAB) responsible of driving malolactic fermentation (MLF) in wine. The key biochemical stage of MLF is the microbial decarboxylation of l-malic acid that leads to the production of l-lactic acid and CO_2_. MLF leads to a decrease in wine acidity and an improved microbial stability and sensorial quality (1–3). Increasing attention to the selection of autochthonous microbes from spontaneous fermentation is warranted to aid the design of specific starter cultures used in fermented foods and beverages with a geographical indication status (4–6). This is particularly true for the grape/wine environment, in which a relationship exists among cultivars, vintages, climates, and wine grape microbial biogeography, suggesting a possible dimension of the so-called microbial terroir (7, 8).

While the only fully complete genome sequence is available for the *O. oeni* PSU-1 strain (9), an increasing number of *O. oeni* assembled genome sequences also have been deposited in the GenBank database (10–12). Moreover, for *O. oeni* strain ATCC BAA-1163, a proteome reference map is also available, which is useful for the validation of annotated genes (13).

Here, we present the genome sequences of five *O. oeni* strains (OM22, OT25, OT4, OT5, and OT3) isolated from Nero di Troia wine (a typical Apulian red wine obtained from uva di Troia, an autochthonous Apulian black grape variety) undergoing spontaneous malolactic fermentation (Table 1) (14).

**TABLE 1:** Summary of information for the whole genomes of the five *Oenococcus oeni* strains OM22, OT25, OT4, OT5, and OT3

*O. oeni* strain	G+C content (%)	Genome size (bp)	No. of genes	No. of proteins	Accession no.	No. of contigs
OM22	38	1,862,817	1,895	1,772	JPEK00000000	23
OT25	37.9	1,834,661	1,845	1,731	JPEM00000000	61
OT4	37.9	1,779,962	1,776	1,654	JPEL00000000	55
OT5	37.8	1,767,097	1,775	1,655	JPEJ00000000	60
OT3	37.8	1,769,724	1,780	1,658	JOOH00000000	61

These new assembled complete genomes represent an important opportunity for assessing the molecular basis of (i) some safety aspects (15–17), (ii) tolerance to the harsh wine conditions (18, 19), and (iii) the contribution to wine sensorial quality (2, 3, 20, 21). To the best of our knowledge, it is the first time that five strains isolated from the same terroir are sequenced.

Two micrograms of genomic DNA was subjected to library preparation using the TruSeq DNA sample prep kit FC-121-1001, according to the manufacturer’s instructions. Whole-genome sequencing was performed using the Illumina GAIIx platform. Prior to assembly, raw reads were filtered using the PrinSeq version 0.20.3 software (22) to remove low-quality 3′ ends (*Q* < 25), reads containing a percentage of uncalled bases (Ns) of ≥10%, and duplicated sequences. The genome sequences of *O. oeni* OM22 were *de novo* assembled using the Ray version 2.2.0 assembly program (23), with a *k*-mer size of 71. The genome sequences of *O. oeni* OT25, *O. oeni* OT4, *O. oeni* OT5, and *O. oeni* OT3 were *de novo* assembled using CLC Genomics Workbench 7.0, with a *k*-mer size of 64. The sequence was annotated by the National Center for Biotechnology Information (NCBI) Prokaryotic Genomes Annotation Pipeline. The genome information for each strain is summarized in Table 1.

### Nucleotide sequence accession numbers.

The draft genome sequences of the *O. oeni* strains sequenced in this study have been deposited as whole-genome shotgun projects at DDBJ/EMBL/GenBank under the accession numbers JPEK00000000 (*O. oeni* OM22), JPEM00000000 (*O. oeni* OT25), JPEL00000000 (*O. oeni* OT4), JPEJ00000000 (*O. oeni* OT5), and JOOH00000000 (*O. oeni* OT3).
